# A29 INVESTIGATING THE IMPACT OF PARKINSON’S DISEASE-ASSOCIATED GENES ON INTESTINAL HOMEOSTASIS

**DOI:** 10.1093/jcag/gwad061.029

**Published:** 2024-02-14

**Authors:** J Pei, S Recinto, A MacDonald, A Kazanova, C Gavino, L Trudeau, M Desjardins, J Stratton, S Gruenheid

**Affiliations:** . McGill University, Montreal, QC, Canada; . McGill University, Montreal, QC, Canada; . McGill University, Montreal, QC, Canada; . MIM, McGill University, Montreal, QC, Canada; . McGill University, Montreal, QC, Canada; . Universite de Montreal, Montreal, QC, Canada; . Universite de Montreal, Montreal, QC, Canada; . McGill University, Montreal, QC, Canada; . McGill University, Montreal, QC, Canada

## Abstract

**Background:**

Intestinal epithelial cells (IECs) provide an essential physical barrier between harsh luminal contents and underlying host tissue. The maintenance of intestinal homeostasis must be intricately regulated through the proliferation and differentiation of intestinal stem cells (ISCs). Dysregulation of this system results in the loss of barrier function, causing pathologies in both intestinal and extra-intestinal diseases. While Parkinson’s Disease (PD) is primarily a neurodegenerative disorder, there is increasing evidence linking PD progression and gastrointestinal (GI) dysfunction. Constipation and increased bowel permeability are often observed years prior to development of motor dysfunction in PD, and people with inflammatory bowel disease are more likely to develop PD. Our group developed a model to investigate the role of the gut in PD, demonstrating that mice with genetic ablation of the PD-associated gene *Pink1* exhibited motor phenotypes only when previously infected with Gram-negative *Citrobacter rodentium* intestinal bacteria. As *Pink1* and other PD-associated genes are expressed in IECs, we hypothesize that PD-associated gene mutations directly affect the epithelium and impact early PD pathophysiology.

**Aims:**

1. Determine how *Pink1* mutation affects the transcriptional profiles in IECs at steady state and *in vivo C. rodentium* infection.

2. Use colonoid systems to study the effect of *Pink1* mutation on epithelial activity.

**Methods:**

ScRNAseq was performed on IECs isolated from uninfected and infected *Pink1* WT and KO mice, sacrificed at day 7 and 14 (early and peak infection) to elucidate transcriptional differences between epithelial lineages of each genotype. Additionally, colonoids were derived from primary mouse tissue and treated with lipopolysaccharide (LPS) to determine how *Pink1* KO affects the inflammatory response of the epithelium.

**Results:**

Our data revealed that *Pink1* KO profoundly affected several epithelial lineages. ISCs from infected *Pink1* KO mice had dysregulated cell cycle genes, transit amplifying cells showed dysregulated expression of tight junction genes, and enterocytes displayed dysregulation in oxidative damage and apoptotic genes. Preliminary data from colonoids showed that *Pink1* KO mice, when stimulated with LPS, had altered pro-inflammatory cytokine gene expression. We are also evaluating colon motility differences between genotypes through transit time assays, counting fecal pellets per hour, and fecal water content.

**Conclusions:**

In *Pink1* KO IECs, there is indeed an altered cellular response upon infection, but more information is needed to decern the mechanistic role of IECs in PD. By investigating the role of PD genes in the GI tract, these studies carry important implications for understanding the initiation and progression of PD.

**Funding Agencies:**

CIHR

